# A First Insight into the Structural and Functional Comparison of Environmental Microbiota in Freshwater Turtle *Chinemys reevesii* at Different Growth Stages under Pond and Greenhouse Cultivation

**DOI:** 10.3390/microorganisms8091277

**Published:** 2020-08-21

**Authors:** Aiguo Zhou, Shaolin Xie, Di Sun, Pan Zhang, Han Dong, Zhiheng Zuo, Xiang Li, Jixing Zou

**Affiliations:** 1 Joint Laboratory of Guangdong Province and Hong Kong Region on Marine Bioresource Conservation and Exploitation, College of Marine Sciences, South China Agricultural University, Guangzhou 510642, China; aiguozhou@scau.edu.cn (A.Z.); xieshaolin@scau.edu.cn (S.X.); sundi@stu.scau.edu.cn (D.S.); 2@stu.scau.edu.cn (P.Z.); donghan@stu.scau.edu.cn (H.D.); 1808546896@stu.scau.edu.cn (Z.Z.); 2 Guangdong Laboratory for Lingnan Modern Agriculture, South China Agricultural University, Guangzhou 510642, China; 3 Canadian Food Inspection Agency, 93 Mount Edward Road, Charlottetown, PE C1A 5T1, Canada; sean.li3@canada.ca

**Keywords:** *Chinemys reevesii*, different growth stages, pond cultivation, greenhouse cultivation, environmental microbiota

## Abstract

The microbial community structure of water is an important indicator for evaluating the water quality of the aquaculture environment. In this study, the investigation and comparison of the bacterial communities of pond cultivation (PC) and greenhouse cultivation (GC) between hatchling, juvenile, and adult growth stages of *C. reevesii* were performed. In addition, the V4 regions of the 16S rRNA gene were sequenced. The Chao1 richness estimator of the PC group was significantly higher than that of the GC group. The beta diversity showed that the microbiotas of the two groups were isolated from each other. The dominant phyla were Cyanobacteria, Proteobacteria, Actinobacteria, Bacteroidetes, Verrucomicrobia, and Planctomycetes in the PC group and Proteobacteria, Bacteroidetes, Firmicutes, Cyanobacteria, Chloroflexi, and Actinobacteria in the GC group. Both the numbers and the types of Kyoto Encyclopedia of Genes and Genomes (KEGG) pathway annotations differed between the PC and GC groups. The prediction of bacterial phenotype implied that the GC environment is more likely to deteriorate, and turtles are more susceptible to pathogens than those of the PC environment. In addition, a total of nine potential pathogenic bacteria were identified and the correlation of environmental factors analyses showed significant differences of bacterial species between the PC and GC groups, while the potential pathogenic bacteria showed significant correlation with the stocking density, temperature, pH, orthophosphate (PO_4_-P), and dissolved oxygen (DO) in both the PC and GC groups. Noticeably, this is the first report to describe the different microbiota characteristics of the different cultivation environments in the different growth stages of *C. reevesii*, which will provide valuable data for water quality adjustment, disease prevention, and the healthy breeding of turtles.

## 1. Introduction

As a distinctive cultivation model with thousands of years, pond cultivation has an irreplaceable status, while the greenhouse cultivation has become more and more popular [1,2,3,4]. Therefore, comparing the differences of microbial community structures in the aquaculture environment is of great significance for guiding efficient and healthy cultivation. There are about 220 species of turtle in the world, and 15% of them are distributed in China [5]. The breeding of *Chinemys reevesii* has been a growing trend because of its high-quality variety with edible and medicinal value [6]. The production of turtles is comprised of 50,000 tons per year only in China, and the highest yield is the *C. reevesii* [7]. In recent years, the *C. reevesii* has become a fast-rising wave of breeding for more and more turtle farmers. At the same time, a series of breeding models have been established, and a large number of large-scale breeding farms have been formed, of which pond and greenhouse cultivation have become the mainstream and the largest breeding modes [8,9,10]. With the expansion of breeding density, the breeding environment has been under tremendous pressure, which has led to the deterioration of farming water quality, disease outbreaks, drug abuse, and has caused huge losses to the turtle industry as well threatened food quality and safety and human health.

As a variable-temperature animal, *C. reevesii* has strict requirements on the environment; hence, a suitable environment is extremely important for the growth, reproduction, and quality of cultured turtles [11]. However, the living environments of *C. reevesii* are quite different between pond and greenhouse farming [12,13]. The characteristics of the pond cultivation model are relatively low density, low mortality, and ecology, but the changes of temperature are large between day and night or seasons, the controllability is poor, and the growth is slow, but a high quality of commodity turtle will be finally obtained. On the contrary, the cultivation mode of the greenhouse environment has an extremely high density, small temperature change, fast growth, and strong controllability, but the mortality rate is relatively high, the pollution is more serious, and the quality of commodity turtle is lower. Meanwhile, with the continuous expanding of environmentally microbial research, the high-throughput 16S rRNA gene amplicon sequencing was widely used for understanding the effects of a variety of environments on the microbes and also helps to profile complex microbial communities and the diversity of bacterial structure and function with the significant reduction in sequencing costs, which results in a surge of microbial sequencing researches [14,15,16,17,18,19]. The aquaculture systems are complex and easily affected by natural environment and human factors [20,21,22], and the microbial diversity is greatly affected by aquaculture water quality factors [23], for example, the ammonia nitrogen (AN) and total nitrogen (TN) contents had a great influence on the bacterial communities of *Litopenaeus vannamei* culture ponds [24,25]. Hence, the water quality factors, aquatic microorganisms, and the health of aquatic product are closely related to each other.

In this study, the high-throughput Illumina sequencing was used to investigate the water microbial communities in the different breeding periods between pond and greenhouse cultivation of *C. reevesii*. The ultimate goal is to provide novel information about regulating the water quality indices and microflora in aquaculture environments, to provide some guidance for disease prevention, and the healthy breeding of turtles.

## 2. Materials and Methods

### 2.1. Sample Collection and Detection of Water Quality Indices

In total, 11 water samples of ponds (HTPC1-4, JTPC1-4 and ATPC1-3) and 12 water samples of greenhouses (HTGC1-4, JTGC1-4 and ATGC1-4) were collected from the farms in Huadu (N 23.29, E 113.04) and Zengcheng (N 23.28, E 113.75), Guangdong, China. Twenty liters (L) of each sample was packed in a plastic sealed barrel, recorded the information, and then all the water samples were shipped back to the laboratory with ice packs within four hours or less. Meanwhile, the temperature (temp °C), pH, and dissolved oxygen (DO mg/L) of each pond were measured in situ through a portable water quality detector (HACH SL 1000, Loveland, CO, USA), the transparency (cm) was measured by a transparency board, the stocking density (SD ea/m^2^) was recorded through the farm technicians, and the body weight of *C. reevesii* (*n* = 50) was randomly selected in each pond and was weighed (accurate to 0.01) as well. Additionally, the total ammonia nitrogen (NH_4_-N, mg/L), nitrite nitrogen (NO_2_-N, mg/L), and phosphate (PO_4_-P, mg/L) were determined according to the standard methods of Jin and Tu (1990) [26] within 24 h. Chlorophyll a (μg/L) was determined photometrically after filtration with 0.45 μm membrane using the spectrophotometric method [27] (Sartory and Grobbelaar 1984). The remaining water samples were filtered through 0.22 μm membranes (Sartorius stedimbiotect, Goettingen, Germany) within 24 h, and then stored at −80 °C.

### 2.2. DNA Extraction, Amplicon Generation, Library Preparation and Sequencing

Total genome DNA of 23 samples was extracted using the MOBIO Powerwater^®^ DNA Isolation Kit (MOBIO Laboratories, Carlsbad, CA, USA), 1% agarose gel electrophoresis was used to determine the integrality of the total DNA, and the NanoDrop One (Thermo Fisher Scientific, Waltham, MA, USA) was used to measure the concentration and purity of the total DNA. V4 regions in 16S rRNA genes were amplified using specific primers 515F (5′-GTGCCAGCMGCCGCGGTAA-3′) and 806R (5′-GGACTACHVGGGTWTCTAAT-3′) [28] (Caporaso et al., 2011) with 12 bp barcode. Primers were synthesized by Invitrogen (Invitrogen, Carlsbad, CA, USA). PCR reactions system including: 2x Premix Taq (Takara Biotechnology, Dalian Co. Ltd., China) 25 μL, each primer 1 μL (10 mM) and DNA (20 ng/μL) template 3 μL; then, PCR reactions were performed by a PCR instrument of BioRad S1000 (Bio-Rad Laboratory, Hercules, CA, USA): 5 min at 94 °C for initialization; 30 cycles of 30 s denaturation at 94 °C, 30 s annealing at 52 °C, and 30 s extension at 72 °C; followed by 10 min final elongation at 72 °C. The length and concentration of PCR products were determined by using 1% agarose gel electrophoresis. The PCR products were mixed in equidensity ratios according to the Gene Tools Analysis Software (Version 4.03.05.0, SynGene). Then, the mixture PCR products were purified by E.Z.N.A. Gel Extraction Kit (Omega, GA, USA).

Sequencing libraries were generated using NEBNext^®^ Ultra™ II DNA Library Prep Kit for Illumina^®^ (NewEngland Biolabs, Ipswich, MA, USA) following the manufacturer’s recommendations and index codes were added. The library quality was determined by the Qubit@ 2.0 Fluorometer (Thermo Fisher Scientific, Waltham, MA, USA). At last, the library was sequenced by an Illumina Nova6000 platform and 250 bp paired-end reads were generated according to the manufacturer’s instruction.

#### 2.3. 16S rRNA Gene Sequence Analysis

Firstly, the quality of Raw Reads was controlled using Fastp (version 0.14.1, https://github.com/OpenGene/fastp) by sliding window, and the primers were removed by cut adapt software (version 1.9.2; https://github.com/marcelm/cutadapt/) in order to obtain the Paired-end Clean Reads. Then, the Paired-end Clean Reads were merged using USEARCH fastq mergepairs (V10, http://www.drive5.com/usearch/) according to the relationship of the overlap between the Paired-end Reads, and 5 bp maximum mismatch was allowed in an at least 16 bp overlap region in order to obtain Raw Tags. The clean tags were obtained by Raw Tags filtration using Trimmomatic v0.33 [29]. Then, the chimera sequences were identified and removed using UCHIME v4.2 [30] in order to obtain Effective Tags.

The operational taxonomic units (OTUs) were clustered with 97% similarity by the Qiime 2 standardized process [31] and the Open Reference OTU picking algorithm. Each representative sequence was compared with the Greengenes database (http://greengenes.lbl.gov) for the generation of Biomdata, including sample name, strain annotation and abundance information [32]. The taxonomy results of species annotations are divided into 7 levels: kingdom (L1), phylum (L2), class (L3), order (L4), family (L5), genus (L6), and species (L7) for 16S rRNA amplicon. Additionally, the unassigned and singleton OTU were removed [33,34], and the effective Tag sequence number (No. of seqs) and OTU taxonomy comprehensive information table (OTU table) of each sample were obtained.

### 2.4. Statistical Analysis

The reads and OTUs of each sample were counted, the Pan and Core of target classification level in different sample numbers were counted using R program (V5.1.3) [35]. Alpha-diversity analyses, including the observed species index, Chao1 richness estimator, Shannon–Weiner index, equitability index, Simpson diversity index, Good’s Coverage index and the phylogenetic diversity index, and Beta-diversity measurements, including principal component analysis (PCA), analysis of similarities (ANOSIM) [36], and nonmetric multidimensional scaling (NMDS) [37], were performed to calculate the differences using R program ggplot2 package [38]. The correlation analyses of environmental factors, microbial community structure distribution, LDA Effect Size (LEfSe) and random forest map of various levels of microbial were analyzed using R package software. Phylogenetic Investigation of Communities by Reconstruction of Unobserved States (PICRUSt) [39] was used to predict the microbial community function and its differences; BugBase (https://bugbase.cs.umn.edu/) was used to predict the bacterial phenotype; one way analysis of variance (ANOVA) were performed to analyze the differences between groups using SPSS 17.0 (SPSS Inc., USA). *p* < 0.05 was considered significant, while *p* < 0.001 was considered highly significant, all the values were the average of the different groups (mean ± SD).

## 3. Results and Discussion

### 3.1. Environmental Factors Analyese

Water quality indices are important for evaluating the breeding environment and are also closely related to microbial communities [24,40]. The SD also directly affects water quality [41,42]. We found that the environmental factors with large differences are concentrated in pH, transparency, DO, NH_4_-N, PO_4_-P, chlorophyll a, and SD. By comparing two different farming breeding environments, we can see that the SD, DO, NH_4_-N, and PO_4_-P parameters of the pond cultivation (PC) environment were significantly less than those of the greenhouse cultivation (GC) environment, while the temperature, pH, and transparency were significantly higher than those of the GC environment (Table 1). These findings imply that the PC environment is more natural and ecological than that of the GC environment.

### 3.2. Analyses of Bacterial Diversity

The bacterial 16S rRNA gene V4 regions of pond cultivation (PC) and greenhouse cultivation (GC) in hatchling, juvenile, and adult periods of *C. reevesii* were sequenced using the Illumina Nova6000 platform. A total of 1,993,090 high-quality counts and an average of 86,656.09 counts were obtained from 23 samples belonging to six groups (HTPC1-4, JTPC1-4, ATPC1-3, HTGC1-4, JTGC1-4, and ATGC1-4). All sequences were clustered into 105,159 OTUs in total, and each sample showed a different number of OTUs clustering information ranging from 3193 (HTGC4) to 6411 (ATPC2) at 97% similarity. The counts, observed OTUs, Chao1, Equitability, Shannon, Simpson, Good’s coverage, and phylogenetic diversity (PD) whole-tree statistical estimates of richness and diversity indexes from each sample are presented in Table 2.

**Figure 1:**
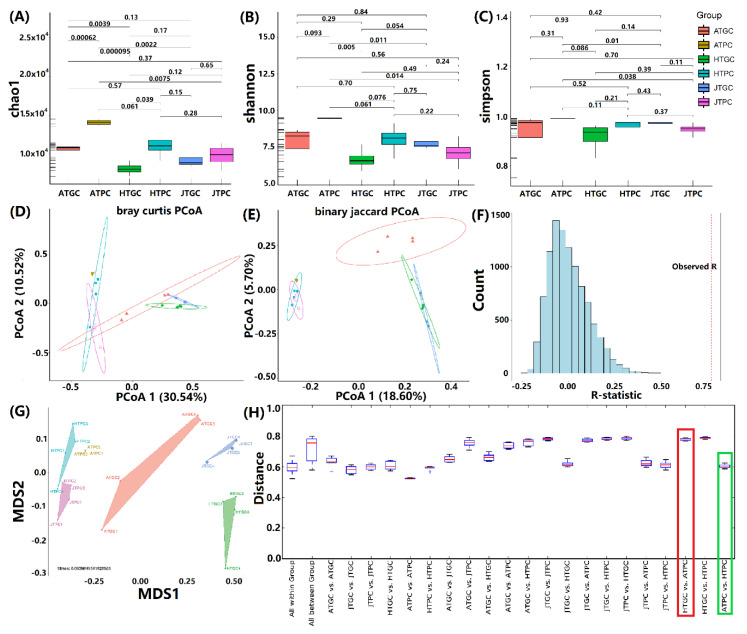
Alpha- and beta-diversity, analysis of similarities (ANOSIM) group similarity, NDMS, and group distance analyses of different growth stages of freshwater turtle *C. reevesii* in six groups. (**A**): Chao1, (**B**): Shannon, (**C**): Simpson, (**D**): Bray–Curtis, (**E**): binary Jaccard, (**F**): ANOSIM group similarity, (**G**): NDMS analysis, (**H**): group distance. The red boxes represent the maximum group distances and the green boxes represent the minimum group distances.

Comparison and analysis of alpha-diversity for different growth stages of freshwater turtle *C. reevesii* in PC and GC environments showed that the total and each group of the Chao1, Shannon, Simpson in PC groups were higher than those of the GC groups (Figure 1A–C). These indicated that the bacterial abundance in pond environment was higher than that of greenhouse environment. Meanwhile, beta-diversity was used to calculate the distance between different groups. The principal component analysis (PCoA) showed that the different growth stages of the GC and PC groups could obviously gather together, but the two cultivation environments are isolated from each other, and the cumulative contribution rate of PCoA 1 (30.54%) and 2 (10.52%) with Bray–Curtis, 18.6% and 5.7% of Binary-Jaccard (Figure 1D,E). Furthermore, the analyses of group differences showed that the observed *r* value is 0.7713 (*p* = 0.0001), and the distance of all within-groups ranged from 0.5211 to 0.6712, and all between-groups ranged from 0.5781 to 0.7977; the mean maximum and minimum group distances between each other were 0.7868 for hatchling turtle of greenhouse cultivation (HTGC) vs. hatchling turtle of pond cultivation (HTPC) and 0.6026 for adult turtle of pond cultivation (ATPC) vs. HTPC. This indicated that there are obvious differences between the groups (Figure 1F, H). The NMDS analysis showed that the microbiota of six groups was clearly separated into six clusters, especially for PC and GC groups (Figure 1G). These results indicated that the microbiota communities of different growth stages, especially in different cultivation environments obvious differences, and the similarity of microbiota types in PC groups was higher than those of the GC groups, and the environment of the PC groups was relatively stable, but the environment of the GC groups was greatly affected by the external environment [43,44]. The Alpha- and beta-diversity of different growth stages of freshwater turtle *C. reevesii* in six groups can be found in Supplementary material Figure S1.

### 3.3. Analysis of Microbiota Structure

**Figure 2:**
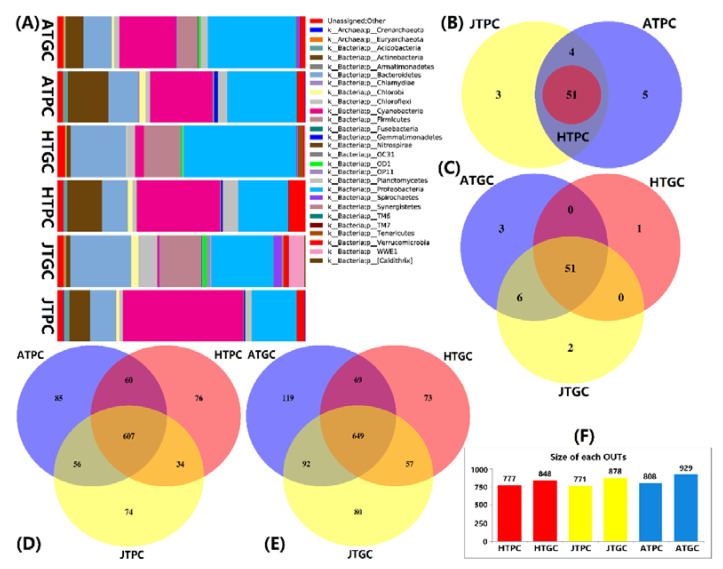
The bacterial community, microbiota structure and OTUs in phylum and genus levels of the pond cultivation (PC) and greenhouse cultivation (GC) groups. (**A**): microbial community structure; (**B**,**C**): Venn diagram of PC and GC groups in phylum level; (**D**,**E**): Venn diagram of PC and GC groups in genus levels; (**F**): Size of each OUTs in HTPC and HTGC, JTPC and JTGC, ATPC and ATGC.

The microbiota of 23 samples (PC 11 and GC 12) was counted. The shared top10 phyla in both PC and GC environments were Proteobacteria (28.7%), Cyanobacteria (22.4%), Bacteroidetes (15.1%), Actinobacteria (8.1%), Firmicutes (6.9%), Chloroflexi (2.9%), Verrucomicrobia (2.9%), Planctomycetes (2.6%), Chlorobi (1.6%), Actinobacteria (1.1%), and WWE1 (1.1%); meanwhile, two Archaea (Euryarchaeota 0.3% and Crenarchaeota 0.1%) were also found in two culture environments (Figure 2A). Furthermore, both the PC and GC models have 63 different prokaryotic phyla and 51 central intersection strains from the 16S rRNA gene sequences. Surprisingly, all the phyla of HTPC groups belonged to JTPC and ATPC groups (Figure 2B,C), which presents the relative ecological stability and regularity of the PC environment [45]. In addition, the top one phylum in PC groups was Cyanobacteria (35.7%), and in GC groups was Proteobacteria (35.4%). These may be closely related to the Cyanobacteria being the dominant algae, which also explain that the oxygen content of the pond is low, resulting in the large multiplication of aerobic bacteria [46]. Conversely, the Proteobacteria is the main source of multiple pathogens with a higher pathogenic potential in the GC environment. Furthermore, the genus levels of PC and GC groups showed that the microbiota of different breeding periods and different breeding modes differed, especially for the GC groups (Figure 2D,E). These indicate that microbiota structures closely related to the breeding environment, feeding method, and management measures [47,48,49]. There were 626 (PC) and 649 (GC) OTUs shared among PC and GC groups, representing 26.6% and 24.4% of total reads separately in PC and GC environments, respectively. In addition, the quantity of OTUs were sorted from ATGC (929) > JTGC (878) > HTGC (848) > ATPC (808) > HTPC (777) > JTPC (771) (Figure 2F). The other taxonomy results of Venn diagrams were provided in the Supplementary material Figure S2.

### 3.4. Annotation Analysis of the Microbiota

**Figure 3:**
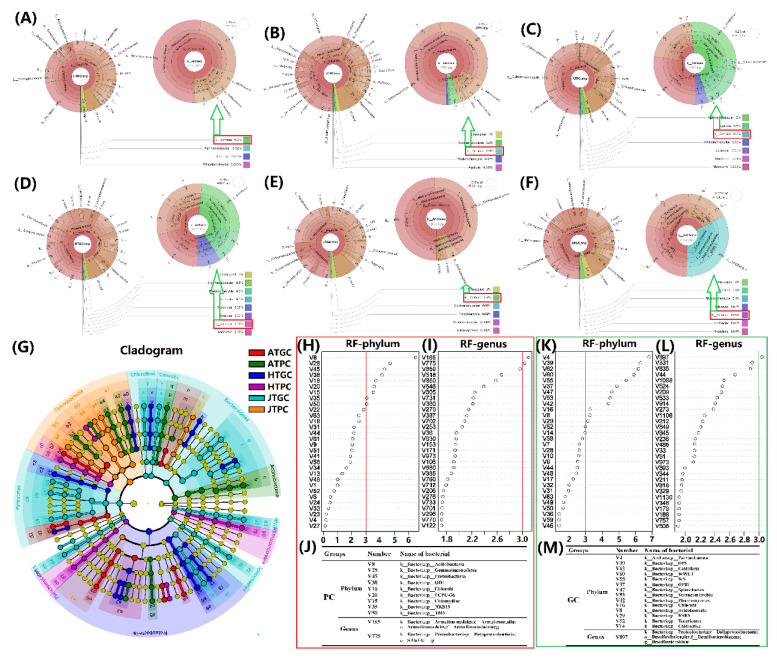
Bacterial and Archaea, LEfSe analysis and random forest model of different classifications annotation of the freshwater turtle *C. reevesii* in six groups. (**A**–**F**): HTPC group, JTPC group, and ATPC group, HTGC group, JTGC group, and ATGC group. The circles represent different classification levels from inside to outside, and the size of the fan represents the relative proportion of different OTU annotation results. The group level was constituted by the average level of relative abundance of each species in each group (*n* = 3 or 4); (**G**): LEfSe cladogram, the circle radiating from the inside to the outside represents the classification level from the phylum to the genus (the innermost yellow circle is the Kingdom). Each small circle at a different classification level represents a classification at their levels, and the diameter of the circle represents the relative abundance; (**H**–**M**): random forest model maps of PC and GC in phylum and genus level based on MDA algorithm.

In order to visualize the annotation results, the KRONA software was used to reveal the average relative abundance of each bacterium in PC (Figure 3A–C) and GC groups (Figure 3E,F). The results showed that the most abundant bacterium was *Cylindrosper-mopsis* of HTPC (11%); *Microcystis* of JTPC (7%); *Synechococcus* of ATPC (11%); *Paludibacter* of HTGC (9%); *Microcystis* of JTGC (20%); *Arcobacter* of ATGC (23%). We can see that the annotated bacteria of different classifications in different growth stages of different culture environments have significant differences, which are extremely consistent with the actual breeding situation [50,51]. Meanwhile, the Archaea in HTPC (0.1%), JTPC (0.06%), and ATPC (0.06%) as well as Euryarchaeota in HTGC (0.8%) and ATGC (0.3%) and the Archaea in JTGC (0.6%) were also found by using KRONA software annotation (Figure 3A–F). The results showed that the richness of archaea in the GC environment was far greater than that of the PC environment. These might be closely related to the water source and the length of aquaculture cycle [52,53,54,55]. In addition, a total of 326 biomarkers with statistical differences were obtained in six groups by using LDA Effect Size (LEfSe) analyses, and the LDA values ranged from 3.3745 to 5.5156, and the relative abundances of microbiota in different growth stages of PC and GC environments showed significant differences (Figure 3G). The mean decrease accuracy (MDA) of random forest model was used to analyze the significant of classification meaning based on the strains with MDA > 3 [56,57]. The results showed that the Acidobacteria, Gemmatimonadetes, proteobacteria, OD1, Chlorobi, FCPU426, Chlamydiae, NKB19, TM6 of the PC groups (Figure 3H–J) and the Parvarchaeota, OP8, Caldithrix, WWE1, WS1, OP11, Spirochaetes, Verrucomicrobia, Planctomycetes, Chlorobi, Acidobacteria, KSB3, Tenericutes, and Caldiserica of the GC groups have the significant meaning for classification at the phylum level (Figure 3K–M). The annotated results demonstrated that the dominant aquatic bacteria in the different growth stages of PC and GC environments changed, which may closely related to the change of the environmental temperature, regulation of water quality, and feeding of bait [58,59,60].

### 3.5. Functional Prediction of the Microbiota

#### 3.5.1. Analyses of Picrust Gene Function Prediction Expression

**Figure 4:**
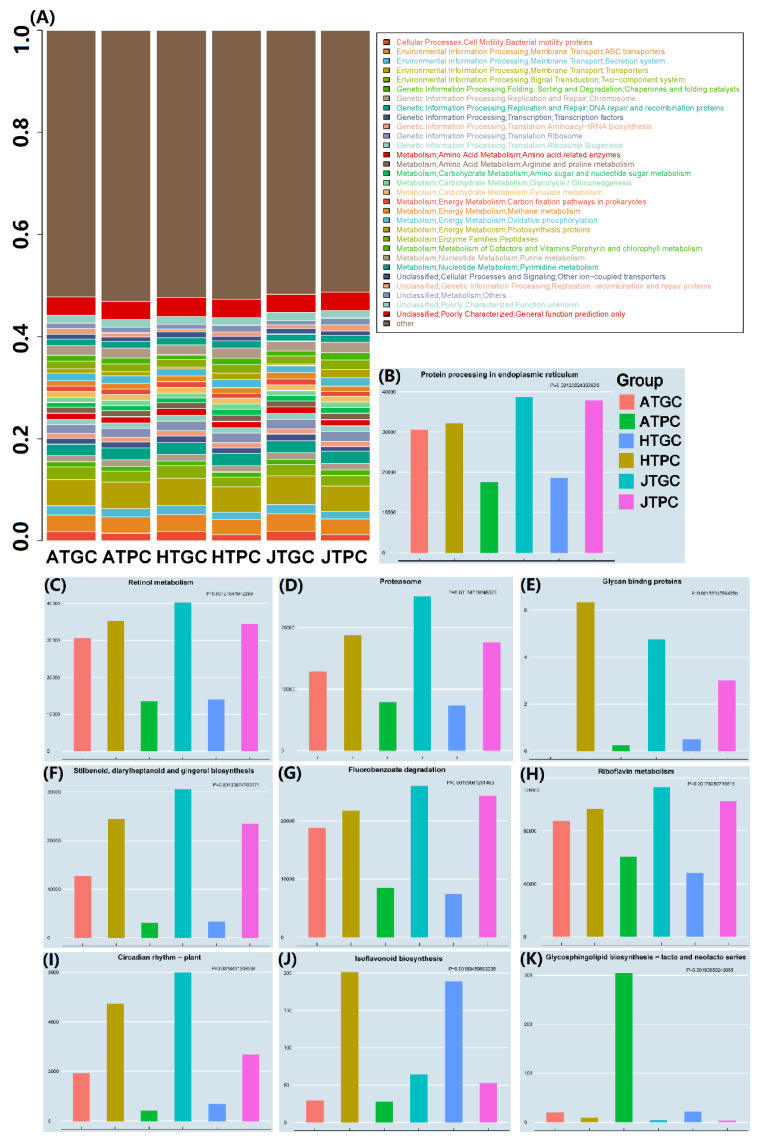
Gene function prediction (top30) (**A**) and top10 in gene function predictive expression *p* value (**B**–**K**) of all six groups based on the COG database.

The Phylogenetic Investigation of Communities by Reconstruction of Unobserved States (PICRUSt) was used to analyze the microbial species composition information based on the 16S rRNA gene sequences [39], the composition of functional gene, and functional differences of different samples in PC and GC environments. Meanwhile, the prediction genes were compared with the COG database, and the results showed that a total of 187 gene functions were predicted, including Cellular Processes (8), Environmental Information Processing (9), Genetic Information Processing (22), Human Diseases (7), Metabolism (113), Organismal Systems (5) and Unclassified (23). Furthermore, the top30 of gene functions were selected, which showed that the most abundances are Bacterial motility proteins in all the growth stages of both PC and GC groups (Figure 4A). As we all know, turtles are fed a large amount of bait during their breeding process, especially the greenhouse cultivation, which greatly accelerates the content of organic matter in the water, and greatly promotes the metabolic function of the water microorganisms [61,62]. In addition, the top10 with the smallest *p* values were obtained according to the information of Picrust prediction gene function using a non-parametric test, including protein processing in endoplasmic reticulum (*p* = 0.001232), retinol metabolism (*p* = 0.001276), proteasome (*p* = 0.001347), glycan binding proteins (*p* = 0.001523), stilbenoid, diarylheptanoid and gingerol biosynthesis (*p* = 0.001638), fluorobenzoate degradation (*p* = 0.001691), riboflavin metabolism (*p* = 0.001783), circadian rhythm-plant (*p* = 0.001843), isoflavonoid biosynthesis (*p* = 0.001895), glycosphingolipid biosynthesis lacto, and neolacto series (*p* = 0.001907). Meanwhile, the gene function expressions prediction of the HTGC, ATPC, and JTPC groups showed relatively higher among the six groups based on top10 Picrust prediction gene functions (Figure 4B–K). These are closely related to the environment of pond farming with easy to break out of Cyanobacteria [63,64]. In addition, both the highest proportion of function annotations in the PC and GC environments were metabolism (51.2% and 57.6%), and the highest proportion of specific functions were concentrated in transporters (5% and 5.3%). These indicated that the prediction functions of the PC and GC environments were similar, but there were differences between the overall levels. The identified bacteria that also related Human Diseases (3.74%) imply potential human pathogens and a certain threat to public health [65].

#### 3.5.2. KEGG Pathway Annotation and Bacterial Phenotype Prediction

**Figure 5:**
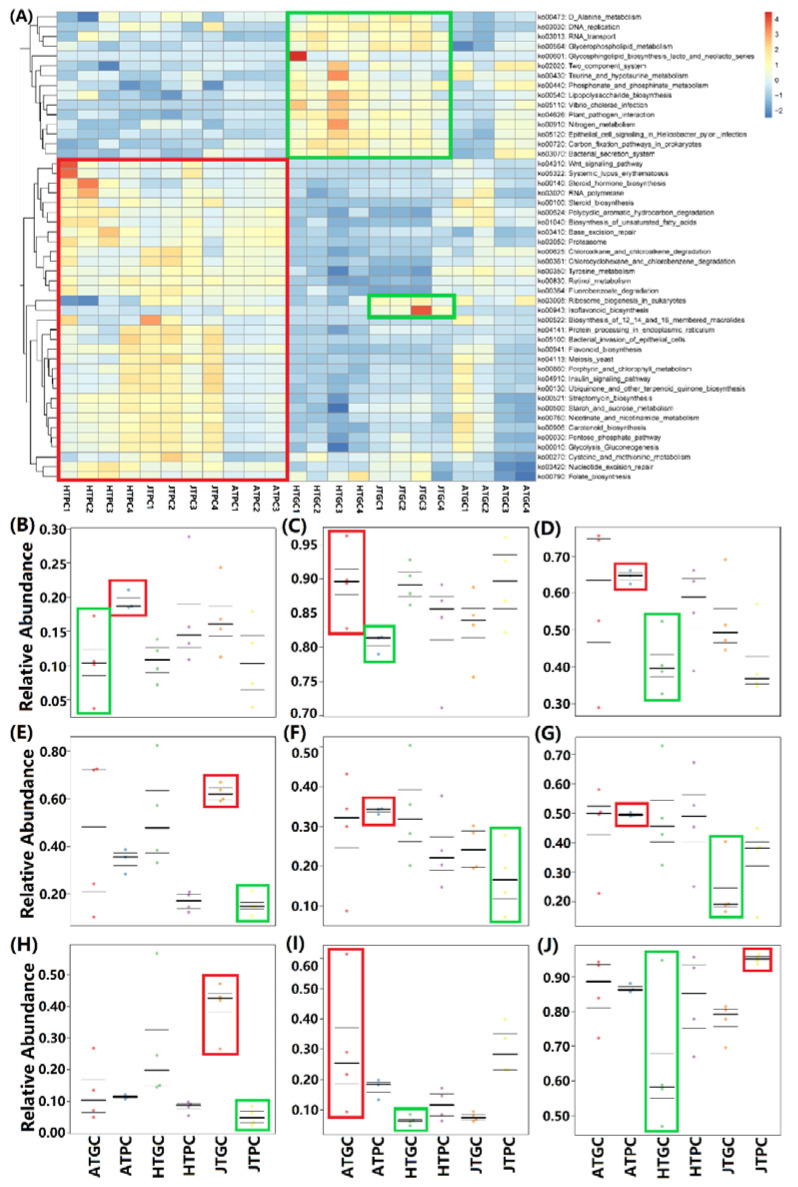
Kyoto Encyclopedia of Genes and Genomes (KEGG) pathway annotation information and prediction of bacterial phenotype of PC and GC groups. (**A**): KEGG pathway annotation, the red and green boxes represent the high abundance of PC and GC environments. The black boxes represent the high abundance of the HTPC group, the blue boxes represent the high abundance of the JTPC group, and the red boxes represent the high abundance of the HTGC group. The green boxes represent the high abundance of the JTGC group, and the gray boxes represent the high abundance of the ATGC group. (**B**): Gram Positive; (**C**): Gram Negative; (**D**): Biofilm Forming; (**E**): Pathogenic Potential; (**F**): Mobile Elements; (**G**): Aerobic; (**H**): Anaerobic; (**I**): Facultative anaerobic; (**J**): Oxidative Stress Tolerant. The red boxes represent the highest relative abundance and the green boxes represent the lowest relative abundance.

The Picrust gene function prediction results were used to enrich the Kyoto Encyclopedia of Genes and Genomes (KEGG) pathway annotation information, and the KEGG pathway annotation heat map with *p* < 0.05 was selected according to the Kruskal test. The original data of heat maps have been standardized and normalized (heat map data = original data-mean/standard deviation). A total of 170 KEGG pathways and 48 significant KEGG pathways were annotated in six groups. There are 15 KEGG pathways showed high relative abundance in the GC groups, especially in the HTGC and JTGC groups, while 31 KEGG pathways showed high relative abundance in the PC groups, especially in the HTPC and JTPC groups (Figure 5A). It can be seen that the signaling pathways of bacteria in the external pond environment are more abundant than those of greenhouse, which may be closely related to sunlight, rainwater, airflow, and soil geology [66,67]. In addition, these results may also be closely related to the difference of growth cycle, SD, water quality, feeding conditions, and environments of *C. reevesii* [68,69].

The bacterial phenotype of all six groups were predicted by using the online tool of BugBase (https://bugbase.cs.umn.edu/) based on the OTUs and mapping files, and the relative abundance of the nine phenotypes (Gram Positive, Gram Negative, Biofilm Forming, Pathogenic Potential, Mobile Elements, Aerobic, Anaerobic, Facultative anaerobic and Oxidative Stress Tolerant) were investigated in this study [70]. We can see that the highest and lowest relative abundance of Gram Positive (mean) were the ATPC and ATGC groups, while the Gram Negative showed the opposite result (Figure 5B,C). The highest relative abundances of biofilm forming, mobile elements, and aerobic were from the ATPC group (Figure 5D,F,G). Both the highest relative abundances of Pathogenic Potential and Anaerobic were JTGC groups (Figure 5E,H). Meanwhile, the highest relative abundances of facultative anaerobic and oxidative stress tolerant were ATGC and JTPC groups (Figure 5I,J). In addition, the lowest relative abundances of biofilm forming, facultative anaerobic and oxidative stress tolerant were from the HTGC group (Figure 5D,I,J). The lowest relative abundances of pathogenic potential, mobile elements and anaerobic were from the JTPC group (Figure 5E,F,H). Finally, the lowest relative abundances of aerobic were from the JTGC group (Figure 5G). These indicated that the relative abundances of Gram positive and aerobic in the pond environment were higher than those of the greenhouse environment, as well as a relatively high oxidative stress tolerance, which was good for the formation of biofilm [71]. Meanwhile, the relative abundances of anaerobic and facultative anaerobic in the greenhouse were significantly higher than those of the pond environment, and an extremely high phenotype of pathogenic potential was found, especially in the JTGC group. These might be related to the enclosed environment, relatively constant temperature, and high breeding density [72,73]. These results also implied that the GC environment is more likely to deteriorate, and turtles are more susceptible to pathogens, while the breeding environment of the pond is more stable and ecological. The statistical test results of BugBase bacterial phenotype prediction can be found in Supplementary material Table S1.

### 3.6. Correlation Analyses of Environmental Factors

**Figure 6:**
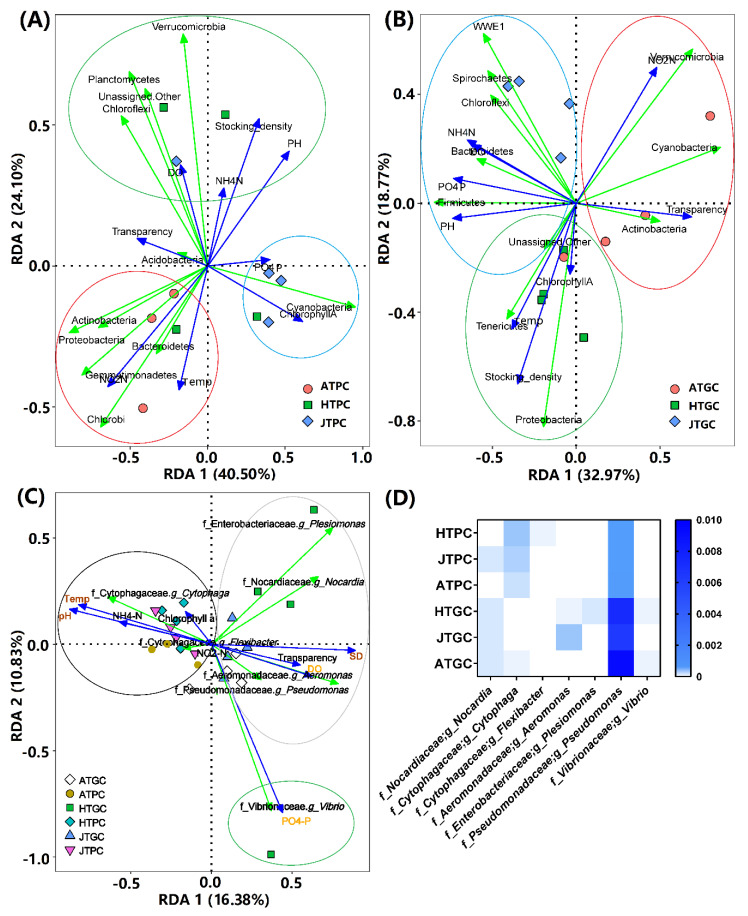
Correlation analysis of environmental factors of PC and GC groups. (**A**,**B**) Redundancy analysis (RDAs) of PC and GC groups based on OTUs of 16S rRNA genes. (**C**): RDA analysis of PC and GC groups based on potential pathogenic bacteria. The blue arrows above represent different environmental factors, and the green arrows represent different phylum of microbiota (top10) and different genus of potential pathogenic bacteria. The arrow length of the environmental factor represents the correlation magnitude between the environmental factors and the samples, and longer arrows represent greater influence on the distribution of the samples and microbiota. The angle between the lines of the arrows represents the correlation. The acute angle indicates that the two environmental factors are positively correlated, and the obtuse angle is negatively correlated. The value in the (**D**) represent the abundance of potential pathogenic bacteria in PC and GC groups.

The relationships between the eight environmental factors and top10 microbiota were determined via redundancy analysis (RDA). The correlation analyses demonstrated that species distribution within the eight environmental factors were 98.79% (PC) and 86.57% (GC). A total of 64.63% (PC) and 51.74% (GC) of the cumulative variances of the microbiota–environment relationship was represented by the first two axes, and the different environmental factors affected the different growth stages samples and top10 microbiota at different levels in both the PC and GC environments. In the PC environment, the plot demonstrated that NO_2_-N (*r* = 0.599, *p* < 0.01) played a significant role in the bacterial community composition (Figure 6A). In the GC environment, pH (*r* = 0.501, *p* < 0.05), PO_4_-P (*r* = 0.505, *p* < 0.05), and SD (*r* = 0.534, *p* < 0.05) played significant roles in the bacterial community composition (Figure 6B). These findings indicate that the environmental factors change with the change in breeding cycle, which affects the growth and living environment of the turtle [74,75]. These findings also explain that the microbial composition and abundance of water are closely related to water quality indicators [25,76].

In addition, a total of four potential pathogenic bacteria in the PC group and six potential pathogenic bacteria in the GC group were identified and three of them were shared in both groups, and the genus *Pseudomonas* showed the most abundance in both PC and GC groups (Figure 6D). Meanwhile, the genus *Myxococcus* was only found in HTPC, and the genera *Aeromonas*, *Plesiomonas*, and *Vibrio* were only found in the GC groups. Furthermore, the correlation of these bacterial genera with environmental factors were also investigated and the results showed that the SD (*p* < 0.001), temp (*p* < 0.001), pH (*p* < 0.001), PO_4_-P (*p* < 0.001), and DO (*p* < 0.001) showed significant correlation with the potential pathogenic bacteria in both PC and GC groups. The correlation analyses showed that the temp, pH, and NH_4_-N showed a positive correlation with the genus *Cytophaga,* while correlating negatively with the other six bacteria. The PO_4_-P showed a positive correlation with the genus *Vibrio* and SD, DO, transparency showed a positive correlation with the genera *Plesiomonas*, *Pseudomonas*, *Nocardia*, and *Aeromonas* (Figure 6C). These findings indicate that the pathogenic bacteria of *Nocardia*, *Cytophaga*, and *Pseudomonas* were common in aquaculture environments [77,78,79], but it was easier to generate and enrich the potential pathogenic bacteria (*Nocardia*, *Cytophaga, Aeromonas, Plesiomonas, Pseudomonas, and Vibrio*) in the GC environment than those in the PC environment (*Nocardia*, *Cytophaga, Pseudomonas, and Flexibacter*). Furthermore, the different environmental factors (e.g., SD, Temp, pH, PO_4_-P, DO, and NH_4_-N) showed significant differences in terms of their impact on potential pathogens [24]. Therefore, balancing and rationally regulating the aquaculture water quality indicators is particularly important for farmers [80,81,82], which also provides important clues for the prevention of aquaculture diseases. The filtered OTU table of potential pathogenic bacteria of PC and GC groups are provided in Supplementary material Table S2.

## 4. Conclusions

Both the pond and greenhouse cultivation play important roles in the development of aquaculture, and they have made tremendous contributions to the production and export of world aquatic products. Therefore, a healthy aquaculture environment is essential to ensure the quality and safety of aquatic products. This study confirms that the microbiota characteristics of different growth stages in pond and greenhouse environments show differences at both the phylum and genus levels, and a certain percentage of archaea was also identified in different growth stages of PC and GC environments. Meanwhile, some potential pathogenic bacteria were also identified both in PC and GC groups and showed different correlations with different environmental factors. The results imply that the water quality of the GC environment is more sensitive to deterioration than the PC environment, and cultured turtles are more susceptible to pathogens, whereas the pond environment is more stable and ecological. These conclusions may be closely related to the unity and controllability of the greenhouse cultivation environment, and we suggest performing ecological breeding methods and developing better tail water treatment technology in order to improve the culture environment as well safe aquatic products. In addition, our study also presents that the high-throughput 16S rRNA gene amplicon sequencing can be used for the identification of bacterial functional diversity and prediction of the potential pathogenic bacteria in the aquaculture environments. Besides, our study also provides new data support for the regulation of the reasonable water quality and diseases prevention in the PC and GC cultured turtles.

## Figures and Tables

**Table 1:** Water quality indices in different periods of pond and greenhouse cultivation of *Chinemys reevesii*.

Samples	HTPC	JTPC	ATPC	HTGC	JTGC	ATGC
Temp (°C)	33.92 ^ABd^ ± 0.10	33.85 ^Ad^ ± 0.06	34 ^Bd^ ± 0.0	31.65 ^Bc^ ± 0.44	30.78 ^Ab^ ± 0.5	30.08 ^Aa^ ± 0.1
pH	9.08 ^ABcd^ ± 0.17	9.48 ^Bd^ ± 0.47	8.57 ^Abc^ ± 0.06	8.12 ^Bb^ ± 0.08	8.1 ^Bb^ ± 0.16	7.28 ^Aa^ ± 0.17
Transparency (cm)	21 ^Ce^ ± 1.41	4.75 ^Ab^ ± 0.29	14.67 ^Bc^ ± 0.58	2.5 ^Aa^ ± 0.58	2.5 ^Aa^ ± 0.56	18 ^Bd^ ± 0.82
DO (mg/L)	4.28 ^Bc^ ± 0.17	1.98 ^Aa^ ± 0.39	2.5 ^Ab^ ± 0.1	7.34 ^Be^ ± 0.06	7.48 ^Be^ ± 0.05	6.43 ^Ad^ ± 0.26
NH4-N (mg/L)	0.25 ^Aa^ ± 0.12	0.75 ^Ba^ ± 0.26	0.46 ^ABa^ ± 0.25	23.14 ^ABab^ ± 20.5	40.06 ^b^ ± 19.9	4.08 ^Aa^ ± 0.26
NO2-N (mg/L)	0.03 ^Aa^ ± 0.03	0.11 ^Aa^ ± 0.09	0.7 ^Bb^ ± 0.05	0.08 ^Aa^ ± 0.04	0.19 ^Ba^ ± 0.04	0.13 ^Aa^ ± 0.01
PO4-P (mg/L)	0.04 ^Aa^ ± 0.03	0.09 ^Aa^ ± 0.09	0.04 ^Aa^ ± 0.03	22.29 ^Bb^ ± 8.78	22.13 ^Bb^ ± 6.18	0.4 ^Aa^ ± 0.1
Chlorophyll a(μg/L)	81.96 ^Aa^ ± 110.08	576.09 ^Bb^ ± 687.06	175.86 ^Aa^ ± 77.54	120.65 ^Ba^ ± 61.35	21.94 ^Aa^ ± 15.78	45.3 ^ABa^ ± 30.19
Stocking density(ea/m^2^)	8.5 ^Cab^ ± 0.58	6.5 ^Bab^ ± 0.58	4.0 ^Aa^ ± 0.00	300 ^Cd^ ± 4.2	59.7 ^Bc^ ± 1.71	9.5 ^Ab^ ± 0.58
Body weight (g/ea)	12.2 ^Aa^ ± 0.21	368.5 ^Bb^ ± 19.87	1695.7 ^Cc^ ± 50.33	7.6 ^Aa^ ± 0.19	208.6 ^Bb^ ± 12.08	1357.1 ^Cc^ ± 37.69

**Note:** Superscript capital letters indicate significant differences in the same farming model in different periods, and lowercase letters indicate significant differences in all six groups. HTPC: hatchling turtle of pond cultivation; JTPC: juvenile turtle of pond cultivation; ATPC: adult turtle of pond cultivation; HTGC: hatchling turtle of greenhouse cultivation; JTGC: juvenile turtle of greenhouse cultivation; ATGC: adult turtle of greenhouse cultivation.

**Table 2:** Richness and diversity indexes relative to each sample (operational taxonomic units (OTUs) were defined at the 97% similarity level (Threshold is 0.03)).

Sample ID	Counts	Alpha Diversity						
		Observed OTUs	Chao1	Equitability	Shannon	Simpson	Goods Coverage	PD Whole Tree
HTPC1	64,997	4825	11,095.6503	0.65086124	7.964142	0.9648892	0.92931319	398.22413
HTPC2	68,075	5884	13,892.7109	0.72650334	9.09769732	0.98811813	0.91357092	458.74608
HTPC3	61,492	5094	10,946.0206	0.66704508	8.21438221	0.97094908	0.92669342	418.07747
HTPC4	91,084	3779	9148.1476	0.56251041	6.6847549	0.93072839	0.94304933	338.30432
JTPC1	88,505	4507	10,507.5396	0.59343605	7.20309818	0.95088012	0.93311305	393.27832
JTPC2	75,770	3969	9333.80887	0.5790958	6.92283544	0.9482456	0.94080717	354.25884
JTPC3	74,256	5068	11,394.1533	0.6663094	8.20040355	0.97460536	0.92570215	417.67826
JTPC4	81,081	3264	7848.80331	0.50961591	5.94845363	0.91409333	0.95031862	310.18648
ATPC1	57,370	6351	14,113.0676	0.74705529	9.4373762	0.99139504	0.90811895	487.5006
ATPC2	67,386	6411	14,508.5938	0.7503018	9.48856696	0.99136054	0.90644324	512.41029
ATPC3	68,135	6240	13,547.0426	0.74615905	9.40707362	0.99027745	0.91149398	496.93705
HTGC1	11,5451	4327	9133.16981	0.6327939	7.64361337	0.96224922	0.93924947	373.40055
HTGC2	89,853	3496	7863.80534	0.56062139	6.59934881	0.92033243	0.94949257	327.23926
HTGC3	88,092	3601	8226.01093	0.49108532	5.80177125	0.83141427	0.94680198	329.16926
HTGC4	10,7919	3193	7111	0.55434821	6.45299941	0.95083458	0.95374085	281.35905
JTGC1	83,718	3699	8325.71587	0.62528623	7.41146738	0.97184881	0.94713241	323.67024
JTGC2	108,954	3940	8986.31754	0.62914851	7.51453719	0.96952937	0.94337975	354.61208
JTGC3	99,788	3933	8727.00998	0.6344613	7.5763653	0.97553313	0.94333255	344.44308
JTGC4	93,833	5140	11,155.2791	0.70375189	8.67553846	0.98252414	0.9279679	406.05474
ATGC1	115,298	3657	9607.0592	0.45725022	5.41221709	0.75224964	0.94399339	364.64287
ATGC2	99,145	4728	10,791.9607	0.65504928	7.99619596	0.96793801	0.93186217	421.06455
ATGC3	82,261	4925	10,803.0905	0.70347102	8.62871079	0.98902736	0.9308945	405.30658
ATGC4	110,627	5128	11,262.0013	0.68522241	8.44480472	0.98016172	0.92763748	407.46934

**Note:** HTPC: hatchling turtle of pond cultivation; JTPC: juvenile turtle of pond cultivation; ATPC: adult turtle of pond cultivation; HTGC: hatchling turtle of greenhouse cultivation; JTGC: juvenile turtle of greenhouse cultivation; ATGC: adult turtle of greenhouse cultivation.

## Supplementary Materials

The following are available online at https://www.mdpi.com/2076-2607/8/9/1277/s1. Figure S1: Alpha- and beta-diversity of different growth stages of freshwater turtle *C. reevesii* in six groups. A: Observed OTUs; B: Equitability; C: Goods coverage; D: PD whole tree; E, F: (PCoA 1, PCoA 3) and (PCoA 2, PCoA 3) of bray Curtis; G, H: (PCoA 1, PCoA 3) and (PCoA 2, PCoA 3) of binary jaccard; I-K: (PCoA 1, PCoA 2), (PCoA 1, PCoA 3), and (PCoA 2, PCoA 3) of weighted unifrac; L-N: (PCoA 1, PCoA 2), (PCoA 1, PCoA 3), and (PCoA 2, PCoA 3) of unweighted unifrac. Figure S2: The bacterial community in class, order, family, and species levels of the PC and GC groups. A-D: Venn diagram of PC groups; E-H: Venn diagram of GC groups. Table S1: The statistical test results of BugBase bacterial phenotype prediction. Table S2: The abundances of potential pathogenic bacteria of PC and GC groups based on filtered OTU tables.
