# Supplementary material for: A First Insight into the Structural and Functional Comparison of Environmental Microbiota in Freshwater Turtle Chinemys reevesii at Different Growth Stages under Pond and Greenhouse Cultivation
Source: Microorganisms. 2020 Aug 21;8(9):1277. doi: 10.3390/microorganisms8091277 (PMC7564371; doi:10.3390/microorganisms8091277)
Supplement: Supplementary file 1 [file microorganisms-08-01277-s001.pdf]

Supplementary data

A first insight into the structural and functional comparison of environmental microbiota in fresh water turtle *Chinemys reevesii* at different growth stages under pond and greenhouse cultivation

Aiguo Zhou[1,2,3#], Shaolin Xie[1,2#], Di Sun[1], Pan Zhang[1], Han Dong[1], Zhiheng Zuo[1], Xiang Li[3], Jixing Zou[1,2*]

[1]Joint Laboratory of Guangdong Province and Hong Kong Region on Marine Bioresource Conservation and Exploitation, College of Marine Sciences, South China Agricultural University, Guangzhou 510642, China

[2]Guangdong Laboratory for Lingnan Modern Agriculture, South China Agricultural University, Guangzhou 510642, China

[3]Canadian Food Inspection Agency, 93 Mount Edward Road, Charlottetown, PEI C1A 5T1, Canada

[*] Corresponding author:

Jixing Zou, E-mail: zoujixing@scau.edu.cn.

[#] These authors contributed equally to this work.

Content: Supplementary data includes two figures and two tables in Page S2-S5:

Fig. S1. ***Page S2***
Fig. S2. ***Page S3***
Table S1. ***Page S4***
Table S2. ***Page S5***

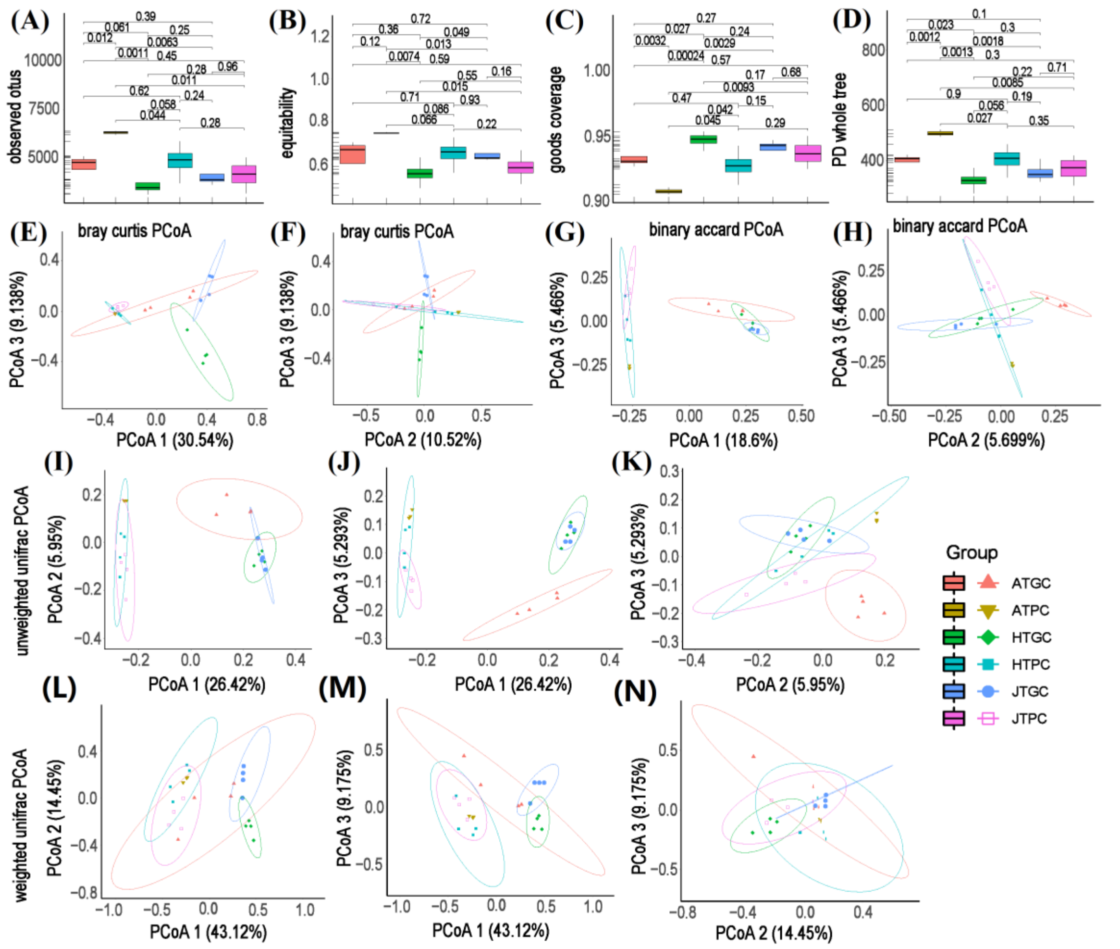

Fig. S1. Alpha- and beta-diversity of different growth stages of freshwater turtle *C. reevesii* in six groups. A: Observed OTUs; B: Equitability; C: Goods coverage; D: PD whole tree; E, F: (PCoA 1, PCoA 3) and (PCoA 2, PCoA 3) of bray Curtis; G, H: (PCoA 1, PCoA 3) and (PCoA 2, PCoA 3) of binary jaccard; I-K: (PCoA 1, PCoA 2), (PCoA 1, PCoA 3), and (PCoA 2, PCoA 3) of weighted unifrac; L-N: (PCoA 1, PCoA 2), (PCoA 1, PCoA 3), and (PCoA 2, PCoA 3) of unweighted unifrac.

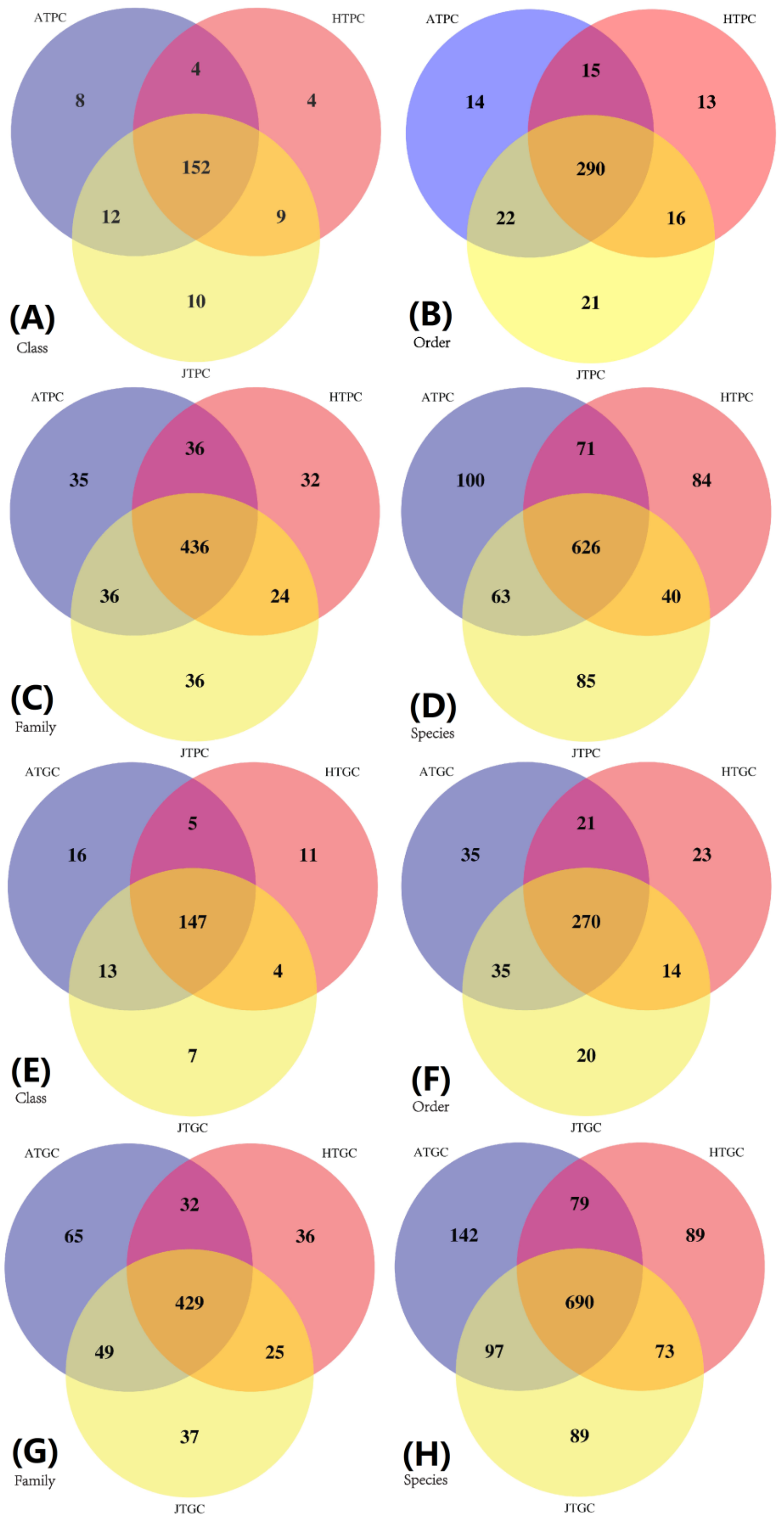

Fig. S2. The bacterial community in class, order, family, and species levels of the PC and GC groups. A-D: Venn diagram of PC groups; E-H: Venn diagram of GC groups.

Table S1. The statistical test results of BugBase bacterial phenotype prediction.

**Group**	**Aerobic**	**Anaerobic**	**Contains Mobile Elements**	**Facultatively Anaerobic**	**Forms Biofilms**	**Gram Negative**	**Gram Positive**	**Potentially Pathogenic**	**Stress Tolerant**
HTPC1	0.672535	0.090257	0.238905	0.146875	0.662319	0.843702	0.156298	0.20803	0.92579
HTPC2	0.527153	0.097231	0.203894	0.063697	0.633002	0.868055	0.131945	0.143815	0.778165
HTPC3	0.453113	0.082591	0.37729	0.084828	0.547639	0.711651	0.288349	0.194623	0.669494
HTPC4	0.250499	0.054299	0.147608	0.172021	0.389582	0.891431	0.108569	0.121288	0.95627
JTPC1	0.385457	0.033555	0.134324	0.231607	0.347565	0.92635	0.07365	0.145412	0.964737
JTPC2	0.378282	0.024922	0.19631	0.335805	0.380694	0.867421	0.132579	0.146256	0.956389
JTPC3	0.448334	0.083373	0.278145	0.39911	0.570517	0.821563	0.178437	0.211518	0.936445
JTPC4	0.145452	0.062888	0.072796	0.231676	0.356757	0.960989	0.039011	0.105153	0.945901
ATPC1	0.493854	0.113214	0.344982	0.198183	0.662149	0.814993	0.185007	0.38559	0.880728
ATPC2	0.489945	0.106741	0.33115	0.18356	0.648277	0.813652	0.186348	0.354915	0.862885
ATPC3	0.502373	0.120088	0.34323	0.133232	0.625155	0.78968	0.21032	0.282108	0.856568
HTGC1	0.427749	0.244982	0.354929	0.085021	0.388501	0.87852	0.12148	0.57237	0.577252
HTGC2	0.483168	0.14455	0.202241	0.048259	0.523863	0.904581	0.095419	0.330905	0.589148
HTGC3	0.729198	0.150463	0.282427	0.062086	0.327993	0.927953	0.072047	0.382389	0.46977
HTGC4	0.323435	0.567699	0.503835	0.064812	0.404034	0.861917	0.138083	0.825372	0.946667
JTGC1	0.188195	0.418942	0.195579	0.081228	0.513368	0.832349	0.167651	0.670683	0.696159
JTGC2	0.191566	0.471569	0.301501	0.062362	0.472281	0.756643	0.243357	0.591118	0.777901
JTGC3	0.165401	0.430544	0.198641	0.068412	0.445403	0.846755	0.153245	0.638973	0.804421
JTGC4	0.403847	0.26517	0.284454	0.094636	0.691649	0.887716	0.112284	0.599503	0.813811
ATGC1	0.226965	0.070498	0.088202	0.6148	0.29034	0.963005	0.036995	0.101528	0.942782
ATGC2	0.581438	0.049422	0.299435	0.290705	0.525837	0.827598	0.172402	0.242016	0.932768
ATGC3	0.49511	0.267732	0.344688	0.094163	0.744129	0.898633	0.101367	0.726465	0.723343
ATGC4	0.504919	0.135081	0.432387	0.21715	0.756393	0.893727	0.106273	0.721434	0.838609

Table S2. The abundances of potential pathogenic bacteria of PC and GC groups based on filtered OTU tables.

**#OTU ID**	**HTPC**	**JTPC**	**ATPC**	**HTGC**	**JTGC**	**ATGC**
f__Nocardiaceae;g__*Nocardia*	0	0.0000118	0	0.0000118	0.0000118	0.0000118
f__Cytophagaceae;g__*Cytophaga*	0.0000295	0.0000236	0.0000157	0	0	0.0000059
f__Cytophagaceae;g__*Flexibacter*	0.0000059	0	0	0	0	0
f__Aeromonadaceae;g__*Aeromonas*	0	0	0	0.0000059	0.0000295	0
f__Enterobacteriaceae;g__*Plesiomonas*	0	0	0	0.0000118	0	0
f__Pseudomonadaceae;g__*Pseudomonas*	0.000183	0.000218	0.000267	0.007386	0.00636	0.009268
f__Vibrionaceae;g__*Vibrio*	0	0	0	0.0000059	0	0.0000059
